# miR-647 and miR-1914 promote cancer progression equivalently by downregulating nuclear factor IX in colorectal cancer

**DOI:** 10.3892/mmr.2022.12713

**Published:** 2022-04-20

**Authors:** Shaoqing Liu, Dingding Qu, Weiping Li, Chenxiang He, Shisen Li, Guosheng Wu, Qingchuan Zhao, Liangliang Shen, Jian Zhang, Jianyong Zheng

Mol Med Rep 16, 8189–8199, 2017; DOI: 10.3892/mmr.2017.7675

Subsequently to the publication of this paper, an interested reader drew to the authors’ attention that, in the scratch-wound assays shown in Fig. 3A on p. 8195, the data shown for the ‘0 h/NC’ and ‘0 h/miR-1914 antagomir’ data panels appeared to be strikingly similar, such that they may have been derived from the same original source.

The authors have consulted their original data, and realize that the ‘0 h/miR-1914 antagomir’ data panel was inadvertently selected incorrectly for Fig. 3A. The corrected version of Fig. 3, now showing the correct data for the ‘0 h/miR-1914 antagomir’ data panel in Fig. 3A, is shown on the next page. Note that the errors in Fig. 3 did not significantly affect the results or the conclusions reported in this paper, and all the authors agree to this Corrigendum. The authors are grateful to the Editor of *Molecular Medicine Reports* for allowing them the opportunity to publish this corrigendum, and apologize to the readership for any inconvenience caused.

## Figures and Tables

**Figure 3.**
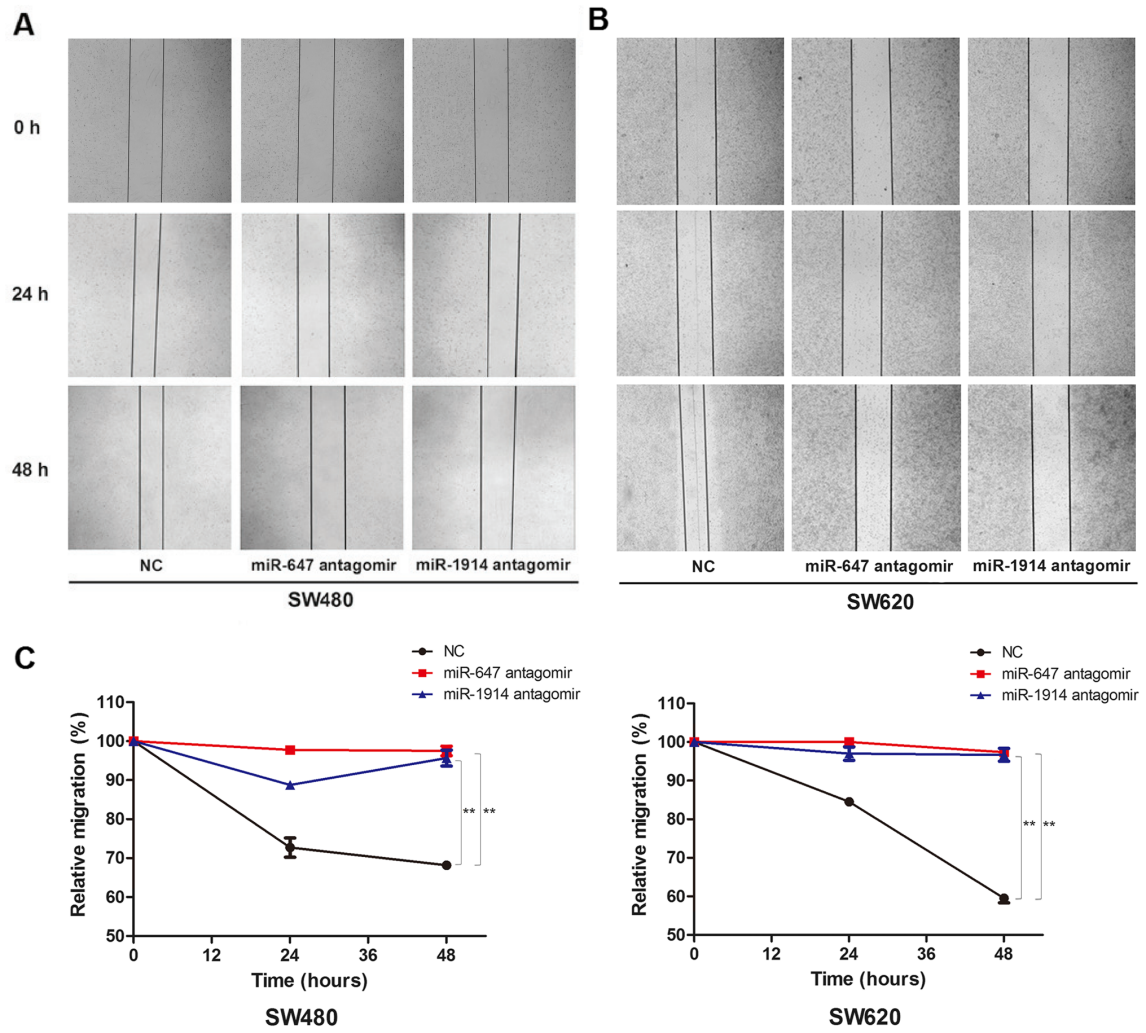
(A) Wound closure of SW480 cells, which were transfected with a miR-647 antagomir or a miR-1914-5p antagomir, in contrast with the NC group, after 0, 24 and 48 h. (B) Wound closure of SW620 cells, which were transfected with a miR-647 antagomir or a miR-1914-5p antagomir, in contrast with the NC group, after 0, 24 and 48 h. (C) The relative migration percentage to quantify the capacity of cell migration among the NC group, miR-647 antagomir group and miR-1914 antagomir group in SW480 and SW620 cells. **P<0.01..

